# Temporal and Spatial Survey on the Abundance of Amoebae and Bacteria in an Estuary and the Role of Environmental Parameters

**DOI:** 10.1111/1758-2229.70198

**Published:** 2025-09-22

**Authors:** Gaëlle Bednarek, Hélène Agogué, Arno Bringer, Tony Agion, Vincent Delafont, Yann Héchard

**Affiliations:** ^1^ Université de Poitiers UMR CNRS 7267, Ecologie et Biologie Des Interactions (EBI) Poitiers France; ^2^ LIENSs Littoral, Environnement et Sociétés, UMR 7266 CNRS‐La Rochelle Université La Rochelle France; ^3^ Qualyse La Rochelle France

**Keywords:** amoeba, bacteria, climate, environmental parameters, estuary, salinity

## Abstract

Free‐living amoebae are phagotrophic protists that prey on bacteria. However, under certain conditions, some bacteria can resist phagocytosis and become pathogenic. Environmental parameters altered by climate change may impact amoebae and bacterial diversity, as well as their pathogenicity. In our study, we monitored amoebae and bacterial abundance both temporally, over 1 year, and spatially, along a river estuary, while also recording key environmental parameters. *Naegleria* was the most represented amoebae genus, present year‐round from the river to the estuary. Similarly, *Vibrio* was the most dominant bacterial genus. Salinity, and to a lesser extent dissolved oxygen, influenced amoebae and bacterial abundance. In particular, the genera *Naegleria*, *Paramoeba*, *Pseudomonas* and *Legionella* were the most affected. In conclusion, this study highlights the impact of salinity on amoebae diversity, suggesting that this parameter, as a key factor in coastal environments, will impact both amoebae and the associated bacterial communities.

## Graphical abstract

Free‐living amoebae and bacteria were monitored along a salinity gradient over 1 year. *Naegleria* and *Vibrio* were the most represented genera, with salinity and dissolved oxygen identified as key factors structuring amoebae diversity and associated bacterial communities under changing environmental conditions. Created in BioRender https://BioRender.com/qcaghmb.

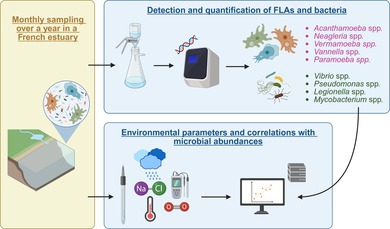

## 1 Introduction

Free‐living amoebae (FLA) are ubiquitous single‐celled organisms, scattered across numerous major eukaryotic lineages, including notably Amoebozoa, Heterolobosea and Rhizaria. They colonise various environments such as soil, seawater and freshwater (Samba‐Louaka et al. 2019; Rodríguez‐Zaragoza 1994). FLA from marine environments are far less described in the literature, but genera largely described in freshwater, such as *Acanthamoeba*, *Vermamoeba* and *Naegleria*, are also found (Samba‐Louaka et al. 2019). Early studies highlighted the frequent colonisation of seawater by FLA, materialised by repeated isolations of *Acanthamoeba*, *Vannella* and *Vexillifera* members (Fernandez et al. 1989). Though the diversity of marine FLA currently remains understudied, it is thought that virtually all clades of amoebae could be found in marine environments (Caron et al. 2012). To support this fact, several studies reported the presence of Vampyrellida FLA (Endomyxa, Rhizaria) in marine environments, where they were shown to significantly impact and regulate blooms of algae and diatoms through their predatory activities (Hess and Suthaus 2022). Among all known marine FLA, members of the *Paramoeba* and *Neoparamoeba* (Dactylopodida, Amoebozoa) genera recently gathered much of the attention (Nowak and Archibald 2018). This is because they are causative agents of the amoebic gill disease, a life‐threatening pathology affecting farmed salmon in marine environments, with great economic implications (Crosbie et al. 2012).

FLA are phagotrophic microorganisms that primarily feed on bacteria, as are all phagotrophic protists. They are thought to regulate microbial populations in the environment (Sherr and Sherr 2002; Pernthaler 2005). Such predatory pressure may have led to the emergence of resistance to phagocytosis, along with an increase in the pathogenicity of resistant bacteria (Cirillo et al. 1994, 1997; Hoque et al. 2022). The repeated observations that bacteria being resistant to FLA can also be resistant to human or animal macrophages tend to support the coincidental evolution hypothesis, according to which virulence can be considered a by‐product of grazing resistance mechanisms (Adiba et al. 2010; Amaro and Martín‐González 2021). As a result, FLA can be considered major contributors to the emergence of new pathogenic bacteria that resist phagocytosis and influence their evolution (Shi et al. 2021).

Among the amoebae resisting bacteria, many are naturally found in freshwater and/or in marine water. It includes bacteria from the following genera: *Legionella* (Rowbotham 1980), *Vibrio* (Van der Henst et al. 2016), *Mycobacterium* (Salah et al. 2009) and *Pseudomonas* (Matz et al. 2008). *Legionella* spp. are ubiquitously found in freshwater in low concentrations as well as in marine water (Borella et al. 2005). *Legionella pneumophila* is mainly found in artificial water systems where the temperature is ideal but could also be detected in marine water in association with FLA and other protists (Gast et al. 2011). *Vibrio* is known for its numerous species described in aquatic environments as both human and aquatic fauna pathogens such as *Vibrio cholerae* or *Vibrio vulnificus* (Chakraborty et al. 1997). *V. cholerae* , the causative agent of cholera, can be isolated from aquatic animals such as crustaceans and fish (Halpern and Izhaki 2017). *Pseudomonas* includes pathogens for humans and animals that can be found in various aquatic environments, such as soil, freshwater and seawater (Mena and Gerba 2009). *Pseudomonas aeruginosa* is a widespread free‐living bacterium that can be found in diverse environments, with its occurrence largely linked to human activities (Crone et al. 2020). Regarding *Mycobacterium*, several pathogenic species for humans and/or animals are present in the marine environment. *Mycobacterium marinum* is mainly responsible for mycobacteriosis in fish, but *Mycobacterium fortuitum, Mycobacterium chelonae* and *Mycobacterium gordonae* can also be involved (Delghandi et al. 2020).

Environmental parameters obviously modify the presence and abundance of microorganisms. Climate change will likely amplify these modifications, and it has been hypothesised that it might increase the development of waterborne (Funari et al. 2012) and marine diseases (Burge et al. 2014). Specifically, it has been suggested that climate change can impact amoeba and hosted bacteria (Heilmann et al. 2024). For example, *Naegleria fowleri*, a thermophilic and pathogenic amoeba, might be favoured by increased temperature, and an increase in *L. pneumophila* cases was linked to rainfall and humidity. Estuaries and coastal waters are highly subjected to environmental parameters such as freshwater inflows, variations in salinity, and temperature fluctuations; they are also among the most degraded ecosystems on Earth (Chilton et al. 2021; Adyasari et al. 2021). Indeed, they are particularly exposed to intense and multiple pressures (Duarte et al. 2015; Jennerjahn and Mitchell 2013), weakening the ecosystem and potentially leading to a loss of biodiversity and ecosystem functions (Cochard et al. 2008). Water quality is also degrading in estuaries and coastal waters, notably due to eutrophication (Howarth et al. 2011; Pinckney et al. 2001) and pollution, but also because of overexploitation (Kennish 2002). These ecosystems are of considerable environmental and economic significance, providing a broad array of goods and services (Luck et al. 2003).

In our study, a temporal (1 year) and spatial (from the river to the estuary at the Atlantic Ocean coast) sampling was performed to assess variations in environmental parameters such as temperature, salinity, dissolved oxygen and precipitations. The presence and abundance of selected FLA, bacterial genera and some pathogenic species were followed by qPCR among these samples. Finally, the correlations between the environmental parameters, the presence of FLA and bacteria were analysed.

## 2 Materials and Methods

### 2.1 Sampling Sites and Environmental Parameters

**FIGURE 1:**
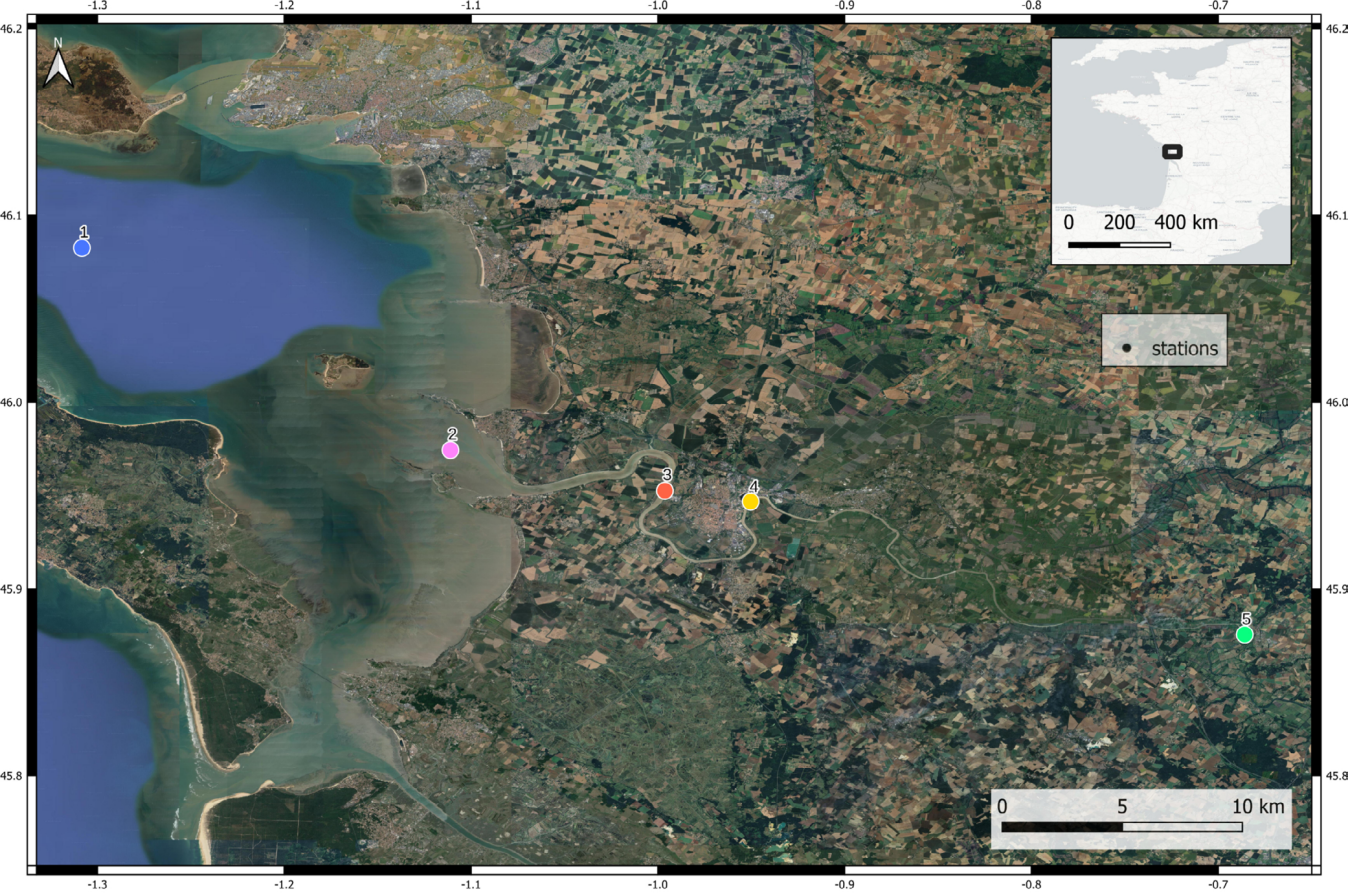
Sampling sites along the Pertuis Charentais area, France.

The Charente is a 350 km coastal river draining a 10,000 km^2^ basin and emerging in the bay of Marennes Oléron (Pertuis Charentais area). The sampling area started at Station 1 within the Pertuis area at 5 nautical miles from the coast and ended at the upstream of the Charente River (Station 5) (Figure 1). The surface water was sampled along a salinity gradient, from marine to freshwaters: Station 1 (46°5.05′ N, 1°18.50′ W), Station 2 (45°58′33.59″ N, 1°6′40.04″ W), Station 3 45°57′15.5″ N, 0°59′46.1″ W), Station 4 (45°56′52.7″ N, 0°56′58.9″ W) and Station 5 (45°52′37.2″ N, 0°41′10.0″ W) (Figure 1). Station 1 was sampled by the French Coastal Environment Monitoring Service (SOMLIT, Philippe et al. 2011); temperature and salinity were measured using a multiparameter probe (Cond 3110, WTW), while oxygen concentration was determined by the Winckler method (Carpenter 1965). Station 2 was sampled by the French Observation and Monitoring program for Phytoplankton and Hydrology in coastal waters (REPHY 2023); temperature, salinity and dissolved oxygen were measured using a multiparameter probe (EXO2, YSI). Finally, Stations 3, 4 and 5 were sampled by the QUALYSE analysis laboratory (La Rochelle, France); temperature, salinity and dissolved oxygen were measured using a multiparameter probe (MultiLine 3630, WTW).

Precipitation data 24 h prior to sampling was collected via the Météo France database. Different weather stations were used for the different sampling sites. For Station 1, weather data were sourced from the weather station at La Rochelle aerodrome (46°10′41″ N, 1°11′35″ W). For Station 2, the data came from the I.N.R.A.E. meteorological station (45°59′18″ N, 1°01′37″ W). Finally, for Stations 3, 4 and 5 along the Charente River, the Saint‐Agnant airfield station (45°53′13″ N, 0°58′56″ W) provided the necessary weather data (Figure S1D). Water samples (2 L) were collected in sterile plastic bottles, on a monthly basis in each station from November 2022 to December 2023. Samples were kept at 4°C until further processing.

### 2.2 Primer Design

Primers targeting the 18S rRNA sequence of *Naegleria*, *Vermamoeba*, *Vannella* and *Paramoeba* genera were designed as follows. Sequences representing each genus were extracted from the GenBank/NCBI database (*Vannella* JQ271763, *Naegleria* M18732, *Vermamoeba* AF426157, *Paramoeba* MG679920) and used to gather homologous sequences using BLAST against the nucleotide database (Altschul et al. 1990), using a query cover threshold of at least 90%. Multiple sequence alignments were performed on NGPhylogeny (Lemoine et al. 2019), using the MAFFT algorithm (Katoh 2002). The resulting alignment was used to design consensus primers on PrimerDesign M (Yoon and Leitner 2015). Default parameters were chosen except for multiple fragments with flex overlap. The candidate primers obtained were then tested for coverage and specificity using the PR2 primer database (Vaulot et al. 2022). In addition to the primers designed for this study, previously published ones were selected from the literature for *Acanthamoeba*, *Vibrio*, *Pseudomonas*, *Legionella* and *Mycobacterium* genera, as well as for each species‐specific target (Table 1). The optimal melting temperature was experimentally determined for each primer pair.

**TABLE 1:** PCR primers used in this study, specific to FLA or bacteria.

	Genus/species	Targets	Primer names	Primer/pro be sequences (5′ → 3′)	Melting temperature (°C)	Coverage	References
FLA	*Acanthamoeba* spp.	18S	AcantF900	CCCAGATCGTTTACCGTGAA	53	95	Qvarnstrom et al. 2006
			AcantR1100	TAAATATTAATGCCCCCAACTATCC			
	*Naegleria* spp.	18S	Nae13‐F	GGAAACTCACCAGGTCAGGACAC	57	95	This study
			Nae13‐R	AAAGGCTTAGGTCTCGTTCGTTATC			
	*Vermamoeba* spp.	18S	Verma10‐F	GGCCTCCTATGTTCCTAACGGTC	57	100	This study
			Verma10‐R	ATGCTAACGTATTCGGAGGCG			
	*Vannella* spp.	18S	Van5‐F	GAGAAGGAGCATGAGAAACG	55	92	This study
			Van5‐R	MTTGTTATTTTTTGTCACTATTTCG			
	*Paramoeba* spp.	18S	Para12‐F	AGAAATCTAGTTCWTTTACTTTGAG	55	97	This study
			Para12‐R	AACTGTCCCTTTTAATCATTACAC			
Bacteria	*Vibrio* spp.	16S	Vib1‐F	GGCGTAAAGCGCATGCAGGT	53		Vezzulli et al. 2016
			Vib2‐R	GAAATT CTACCCCCCTCTACAG			
	*V. cholerae* ^a^	ompW	ompW‐F	TCAATGATAGCTGGTTCCTCAAC	60		Garrido‐Maestu et al. 2014
			ompW‐R	CGATGATAAATACCCAAGGATTGA			
			ompW‐Probe	TGGTATGCCAATATTGAAACAACG			
	*Pseudomonas* spp.	16S	Pse434F	ACTTTAAGTTGGGAGGAAGGG	60		Pereira et al. 2018
			Pse665R	ACACAGGAAATTCCACCACCC			
	*P. aeruginosa*	PA14_4976	PA14F	CGGTACAGGTCGGCACG	55		Wang et al. 2022
			PA14R	CGAGGGACGAAGGTAAGGA			
	*Legionella* spp.^a^	23S	Leg23SF	CCCATGAAGCCCGTTGAA	60		Nazarian et al. 2008
			Leg23SR	ACAATCAGCCAATTAGTACGAGTTAGC			
			Lsp23SP	TCCACACCTCGCCTATCAACGTCGTAGT			
	*L. pneumophila* ^a^	mip	mip_FP	GAAGCAATGGCTAAAGGCATGC	60		Mentasti et al. 2015
			mip_RP	GAACGTCTTTCATTTGYTGTTCGG			
			mip_HP	CGCTATGAGTGGCGCTCAATTGGCTTTA	60		
Bacteria
	*Mycobacterium* spp.^a^	atpE	FatpE	CGGYGCCGGTATCGGYGA			Radomski et al. 2013
			RatpE	CGAAGACGAACARSGCCAT			
			PatpE	ACSGTGATGAAGAACGGBGTRAA			
	*M. marinum* ^a^	erp	MAR‐erpF	TTGGCAGGACGACAAGGTCA	60		Slany 2014
			MAR‐erpR	ATGGTACGAGTGAGGTTGGTGA			
		MAR‐erpPr	TTCGACAACCCAAGCAGGCCCTAAGCA			

^a^ Probe‐based qPCR.

### 2.3 Environmental DNA Isolation

Between 70 and 200 mL of each sample was filtered through a 0.45‐μm‐pore size MF‐Millipore Membrane Filter by vacuum filtration. According to the Power Water DNA Extraction protocol (QIAGEN), filters were inserted into a 5 mL PowerWater Bead Pro Tube with sterile forceps and then stored at −20°C until DNA extraction. Total environmental DNA was extracted from filters using the DNEasy PowerWater extraction kit. DNA extractions were performed with a QIAcube Connect Instrument (QIAGEN), following the manufacturer's recommendations. DNA was eluted in a final volume of 50 μL and stored at −20°C.

### 2.4 Quantitative PCR

All 60 DNA extracts were diluted at 1:4. Quantitative PCR (qPCR) analyses were performed using a LightCycler 480 II device (Roche Diagnostics). qPCR mixes were prepared using Takyon No ROX SYBR 2X MasterMix blue dTTP for primers without probe and Takyon No ROX Probe 2X MasterMix UNG for primers with probe according to manufacturer instructions, in a final volume of 10 μL (Eurogentec). SYBR‐based assays were subjected to the following cycling conditions: initial denaturation at 95°C for 3 min, followed by 45 cycles of 95°C for 3 s, 53°C–60°C for 15 s, according to the primers' melting temperatures (Table 1) and 72°C for 15 s. The melting curve analyses were performed after a denaturation step at 95°C for 5 s, consisting of initial cooling/renaturation at 65°C for 1 min and a gradual increase in temperature until reaching 97°C with a ramp rate of 0.11°C/s and a continuous acquisition of fluorescence. TaqMan‐based assays were subjected to the following cycling conditions: a carry‐over prevention was applied according to the manufacturer's recommendations of 50°C for 2 min, preceding an initial denaturation at 95°C for 3 min (except for *Mycobacterium* spp. and *M. marinum* reactions, which required an initial denaturation of 8 min due to high GC content), followed by 40 cycles of 95°C for 10 s and 60°C for 1 min. The efficiency of all designed primer pairs was assessed through the generation of standard curves. Serial 10‐fold dilutions of the target DNA were amplified. Cycle threshold values were plotted according to the log values of DNA copy number. Slope values were calculated from the obtained standard curves and used to calculate PCR efficiency using the second derivation tool from LightCycler 480 software.

### 2.5 Statistical Analysis

Nonmetric multidimensional scaling (NMDS) analysis was performed using the vegan package version 2.6‐6.1, in RStudio v2024.4.2.764 (Posit team 2024; Oksanen et al. 2024), on qPCR data and environmental data, using Bray–Curtis dissimilarity indices. For each analysis, the number of dimensions (*k*) has been defined: all are represented in two dimensions, that is, *k* = 2. The stress level was considered acceptable if below 0.20. The influence of environmental variables on sample distribution was plotted as vectors, with the significance level corresponding to *p* < 0.05. Correlation analyses were performed using the Corrplot package v 0.95 (Wei and Simko 2024). The Spearman correlation method was employed to explore the relationships between all numeric variables in our dataset. The colour palette was defined using the RColorBrewer package v 1.1.3 (Neuwirth 2022).

## 3 Results

### 3.1 Environmental Characteristics of Sampling Stations

The temporal dynamics of environmental parameters were monitored across all five sampling stations. Salinity assessments revealed a distinct separation between the two coastal water stations and the three estuarine stations (Figure S1A). Although relatively stable, Station 1 exhibited more consistent salinity levels over time compared to Station 2, which displayed fluctuations, including a marked decrease in November 2023, dropping from 34 to 19.3 practical salinity units (psu). Station 3 exhibited pronounced salinity variability, ranging from 0.3 psu in March 2023 to 24.5 psu in July 2023. Similarly, Station 4 also experienced salinity fluctuations, though less pronounced than those observed at Station 3, with values ranging from 0.2 psu in March 2023 to 13.8 psu in September 2023. Both stations followed comparable patterns. No salinity variations were observed at Station 5. Temperature trends were generally consistent across all five stations (Figure S1B). However, the estuarine stations (Stations 3, 4 and 5) were slightly colder in winter and warmer in summer as compared to the marine stations (Stations 1 and 2) (with an average of ±8°C in February 2022 vs. ±23°C in July 2023, Figure S1B).

Dissolved oxygen concentrations exhibited both spatial and temporal variability (Figure S1C). Stations 3, 4 and 5 showed the highest fluctuations over time. At Station 3, dissolved oxygen concentrations ranged from 4.1 mg/L in September 2023 to 12 mg/L in January 2023. Station 4 followed a similar trend with values ranging from 2 mg/L in September 2023 to 11.5 mg/L in January 2023. Station 5 displayed large variations, with peaks ranging from 7.3 to 13.2 mg/L. In contrast, Stations 1 and 2 exhibited greater temporal stability, with only minor fluctuations recorded at Station 2.

Precipitation recorded 24 h prior to the sampling indicated no rainfall at any station in January 2023, February 2023 and June 2023 (Figure S1D). The highest precipitation levels were recorded in November 2023 at Stations 1 and 2, with 12.4 mm and 27.5 mm, respectively. For Stations 3, 4 and 5, the highest recorded precipitation occurred in December 2022, reaching 11.3 mm.

### 3.2 FLA Newly Designed Primers Seem Very Specific

Since genus‐specific primers for FLA were not available for qPCR, we decided to design new primer sets, except for *Acanthamoeba* for which a primer set already exists (Qvarnstrom et al. 2006). These primer sets were tested in silico for their coverage towards the target genus and specificity. To this aim, the PR2 primer database (v.2.0.0) was used allowing a maximum of two mismatches for primer hybridisation (Vaulot et al. 2022). All primer sets were 100% specific towards the target genus or closely related genera (Figure S2). The only exception was that *Vanella* primers hybridised also with *Urocystis* (basidiomycota). Besides, the coverage for each primer set was very high, between 90 and 100% (Table 1 and Figure S2).

### 3.3 Spatial and Temporal Microorganism Abundance

**FIGURE 2:**
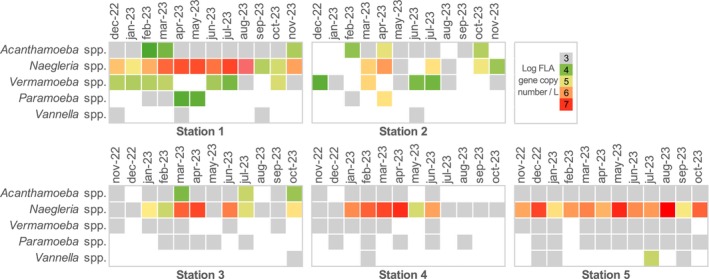
Temporal variation in amoebae abundance across the five sampling stations. Rows correspond to amoebae genera, while columns represent sampling months for each station. The targeted genes are shown in Table 1. Colour variation indicates relative abundance; grey corresponds to values below the limit of quantification.

To follow the presence of five FLA genera and four bacterial genera and their associated pathogen genus, a total of 60 samples were analysed via qPCR (Table 1). All analysed samples were positive for at least one of the FLA monitored with the qPCR assays. Regarding FLA, qPCR analyses showed that the *Naegleria* genus was the most prevalent, being detected in 90% (54/60) of the samples. *Naegleria* was found in all stations, with the highest occurrence in Stations 3, 4 and 5 (Figure 2). Highest *Naegleria* concentrations were detected at Stations 1, 4 and 5. The *Acanthamoeba* genus was detected in 80% of the samples (48/60). However, the samples mostly remained below limits of quantification and were primarily found at Stations 1 and 5. The *Vermamoeba* genus was detected in 65% of the samples (39/60). Like *Acanthamoeba* spp., *Vermamoeba* spp. were predominantly present at Stations 1 and 5, with values below limits of quantification values for the latter. The *Paramoeba* and *Vannella* genera were the least prevalent in the samples, being detected in 43.33% (26/60) and 16.67% (10/60) of the samples, respectively. *Paramoeba* spp. was primarily detected at Station 5 with values that remained below limits of quantification, but the genus was more abundant in both April and May 2023 samples from Station 1. As for *Vannella*, detection occurred sporadically across all monitored stations.

**FIGURE 3:**
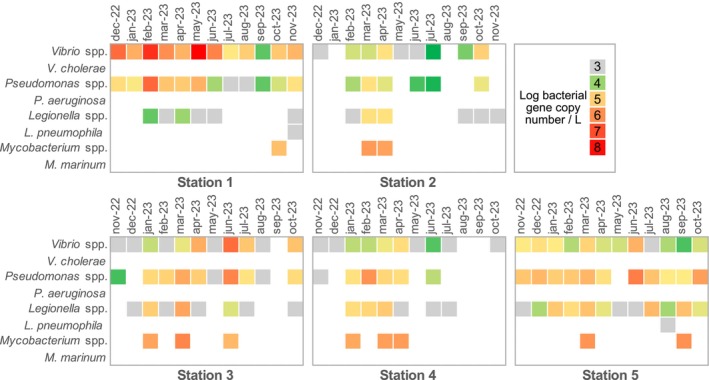
Temporal variation in bacteria abundance across the five sampling stations. Rows correspond to bacterial genera, while columns represent sampling months for each station. The targeted genes are shown in Table 1. Colour variation indicates relative abundance; grey corresponds to values below the limit of quantification.

Bacteria of interest were found in 95% (57/60) of all samples. Among bacterial genera, *Vibrio* and *Pseudomonas* were the most frequently detected, being present in 90% (54/60) and 76.67% (46/60) of all samples, respectively (Figure 3). *Vibrio* reached the highest concentration measured in this study, with a higher abundance at Station 1, peaking in May 2023 at 1.62 × 10^8^ 16S rRNA gene copies/L. *Pseudomonas* spp. was found at all stations, occurring in 76.67% (46/60) of all samples. *Legionella* was omnipresent at Station 5 (freshwater); a decrease in detection frequency was observed along with increasing salinity. The *Mycobacterium* genus was only rarely detected over time, though at all stations. As for the pathogenic bacteria, *V. cholerae* , *P. aeruginosa* and *M. marinum* were not detected in our samples. However, *L. pneumophila* was detected in samples from November 2023 at Station 1 and August 2023 at Station 5, though it remained below limits of quantification (Figure 3).

### 3.4 Impact of Environmental Parameters

**FIGURE 4:**
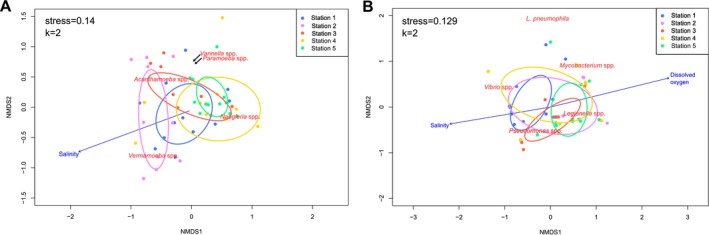
NMDS ordination showing the variations and relationship between water samples based on amoebae (A) or bacterial (B) abundances and environmental factors (temperature, salinity, dissolved oxygen and precipitation). Points represent individual samples. Proximity represents the similarities in microbial community structure and environmental variables. Ellipses represent the distribution of stations, based on the centroids' position with a confidence interval of 95%.

To investigate the potential influence of environmental parameters on microbial presence and abundance, non‐metric multidimensional scaling (NMDS) analyses were performed (Figure 4). Regarding FLA, NMDS ordination based on Bray‐Curtis distances revealed that both sampling stations and salinity were major significant factors shaping amoebae community composition (*p* ≤ 0.001) (Figure 4A). Samples clustered according to their respective stations, indicating a location‐driven structuring of the amoebae community. This suggests that samples from the same station share distinct and similar biological characteristics. Finally, regarding the genera *Paramoeba* and *Vannella*, their abundance was not obviously linked to any station.

For bacterial communities, NMDS analysis using Bray‐Curtis distances identified salinity and dissolved oxygen concentration as the most significant parameters (*p* = 0.017 and *p* = 0.002, respectively) (Figure 4B). The two vectors were opposites, suggesting an inverse relationship between these parameters. While no direct station effect was observed, samples from Station 1 were generally more associated with the salinity vector, whereas those from Station 5 aligned more closely with the dissolved oxygen vector. The abundance of the genus *Mycobacterium* and *L. pneumophila* did not exhibit any apparent association with specific stations or environmental gradients.

**FIGURE 5:**
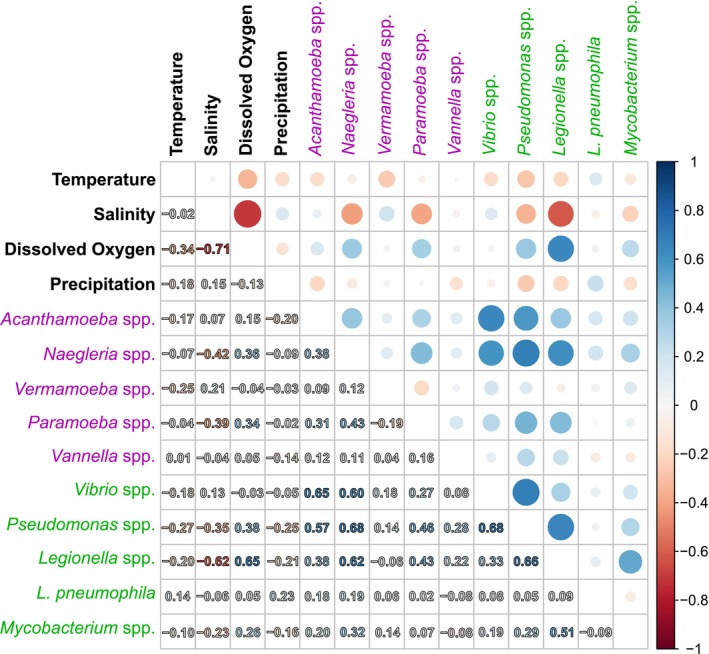
Spearman rank correlation coefficient of microorganisms quantified by qPCR and a selection of measured physicochemical parameters. Red colour denotes negative correlations, while blue colour denotes positive correlations. Size of circles and colour depth represent the correlation strength.

Spearman rank correlation analysis revealed several strong relationships between microbial abundances and environmental factors (Figure 5). The results indicated a significant negative correlation between salinity and both dissolved oxygen (*r* = −0.71) and the *Legionella* genus (*r* = −0.62). Additionally, moderate negative correlations were observed between salinity and the genera *Naegleria* and *Paramoeba* (*r* = −0.42 and r = −0.39). Dissolved oxygen exhibited a strong positive correlation with *Legionella* (*r* = 0.65). Regarding FLA, *Acanthamoeba* showed strong positive correlations with *Vibrio* and *Pseudomonas* (*r* = 0.65 and *r* = 0.57). Similarly, the *Naegleria* genus was positively correlated with the genera *Vibrio* (*r* = 0.60), *Pseudomonas* (*r* = 0.68) and *Legionella* (*r* = 0.62). *Paramoeba* showed moderate positive correlations with *Pseudomonas* (*r* = 0.46) and *Legionella* (*r* = 0.43). In contrast, the genera *Vermamoeba* and *Vannella* did not display strong associations with any parameters. Among bacterial taxa, *Vibrio* demonstrated a strong positive correlation with *Pseudomonas* (*r* = 0.68) while *Pseudomonas* was positively correlated with *Legionella* (*r* = 0.66). Additionally, *Legionella* exhibited a positive correlation with *Mycobacterium* (*r* = 0.51).

## 4 Discussion

Environmental parameters can have a significant influence on microbial populations. This is indeed true for natural variations, as well as human‐induced ones, such as climate change. In our study, we investigated these impacts over a 1‐year period along a river‐estuary continuum. This environmental gradient had a profound influence on various environmental parameters, with salinity standing out as a key factor ranging from 0.2 to 34.87 psu. To our knowledge, this is the first study that follows FLA abundance along such a continuum using qPCR. In this aim, new primers were designed and in silico analysis of the FLA primer sets indicated a combination of high specificity and high coverage, which will allow us to have an accurate quantification of FLA abundance in environmental samples. Although several 18S rRNA gene amplicon sequencing studies have been conducted, FLA were not always effectively amplified with universal primers (Aucher et al. 2020). However, it should be kept in mind that FLA may have high variation in 18S rRNA gene copy number, impairing a precise genus‐level comparison and potentially compromising the quantification accuracy. Despite that, our results demonstrated that FLA were present throughout the study area and across all seasons. Among the detected FLA, it was shown that the genus *Naegleria* is by far the most abundant and frequently occurring. They were present at every station, always in high concentrations, compared to other FLA. Overall, *Naegleria* spp. abundance increased during spring and summer as compared to other seasons. Prior to this study, *Naegleria* has been barely investigated in marine water so far, and only a weak body of evidence supports its presence in high salinity waters (Żbikowska et al. 2013). Such lack of data is partly explained by the fact that much more attention has been directed towards *Naegleria fowleri* , a pathogenic species exclusively found in freshwater (Stahl and Olson 2020). This ecological feature contrasts with other members of the Heterolobosea, which have been repeatedly described in high‐salinity environments (Pánek et al. 2016). By bringing a comprehensive, molecular‐based monitoring along a salinity gradient, our study expands the known ecological range of *Naegleria* spp., suggesting the existence of halotolerant members capable of thriving in high‐salinity conditions.

*Acanthamoeba* and *Vermamoeba* were frequently detected, albeit with distinct seasonal patterns. These two genera were frequently found in aquatic environments, making their presence unsurprising (Khan 2006; Kuiper et al. 2006). Besides, *Paramoeba* and *Vannella*, which are typically associated with marine environments, were not frequently detected, suggesting that their concentration might be below the qPCR detection threshold.

We also monitored bacterial genera that are frequently associated with FLA and related pathogenic species. Not surprisingly, *Vibrio* was detected throughout the study period and across all stations, with the highest abundance recorded at Station 1, which corresponds to marine waters. Indeed, this bacterium is typically found in marine and estuarine environments (Jones 2017). On the contrary, *Mycobacterium* was rarely detected, even in the freshwater stations; but when present, it occurred at relatively high concentrations. Regarding pathogenic bacteria, only *Legionella pneumophila* was detected, appearing in two samples. This species was frequently found in freshwater but not in marine water. However, it has previously been described in marine sediments associated with FLA (Gast et al. 2011). The scarcity of *Mycobacterium* detection in this study may be explained by the fact that only the water column was sampled, and mycobacteria are often found in biofilms in the environment (Esteban and García‐Coca 2018).

NMDS analysis (Figure 4) clearly indicated that salinity is a key driver for FLA as well as for bacteria abundance. Salinity represents the most variable environmental factor in our study, and microbial communities are well known for their sensitivity to salinity fluctuations (Lew et al. 2022). FLA are particularly sensitive to osmotic pressure, and this stress is described to trigger cell differentiation from active trophozoites into a resting cyst form (Fouque et al. 2012). However, some amoebae are known to be resilient to salinity changes, explaining their high presence and abundance in saline environments (Hauer and Rogerson 2005). In addition, dissolved oxygen impacts bacteria abundance, which was not surprising: a previous study has demonstrated that bacterial community diversity and structure are closely linked to dissolved oxygen in coastal waters (Spietz et al. 2015). Indeed, dissolved oxygen in water is generally linked to salinity, but also to temperature and eutrophication (Murray and Riley 1969; Weiss 1970): it has been shown that dissolved oxygen solubility is impacted by salinity, causing a decline as salinity increases (Onabule et al. 2020).

Finally, Spearman rank correlation analysis confirmed this negative correlation between salinity and dissolved oxygen (Figure 5). It also pointed out a significant negative correlation between salinity and *Legionella* and, to a lesser extent, with *Naegleria, Paramoeba* and *Pseudomonas*. *Legionella* is mainly described in fresh water; however, it has recently been identified in multiple sequencing datasets characterising bacterial diversity from marine environments, suggesting that species other than *L. pneumophila* could be naturally found in marine water (Graells et al. 2018). The presence of *L. pneumophila* in marine environments has been linked to anthropogenic sources such as with sewage discharge (Gast et al. 2011), suggesting that it could be transported from the river into estuarine waters. Besides, salinity may impact encystment of FLA, as it is well described that osmotic stress is a key trigger of encystment (Fouque et al. 2012). Interestingly, strong positive correlations were identified between the FLA genera *Acanthamoeba* and *Naegleria* and the bacterial genera *Vibrio*, *Pseudomonas* and *Legionella*, whereas no such correlation was found for the *Mycobacterium* genus. In contrast, *Vermamoeba* and *Vannella* genera did not exhibit strong associations with these bacteria, suggesting different ecological interactions among FLA genera. Positive correlation may indicate either preferential predation of *Vibrio*, *Pseudomonas* and *Legionella* by *Acanthamoeba* and *Naegleria* or, less likely, a mutualistic relationship in which these bacteria benefit from FLA presence to proliferate.

## 5 Conclusion

FLA were detected throughout the year at all sampling sites, with *Naegleria* identified as the most abundant genus. The newly developed primer sets demonstrated strong specificity for genus‐level detection of FLA. Among the bacterial communities, *Vibrio* were the most prevalent, followed by *Pseudomonas*, with minimal detection of pathogenic species. Salinity and dissolved oxygen emerged as the primary environmental factors influencing microbial populations, notably *Naegleria*, *Paramoeba*, *Pseudomonas* and *Legionella*. Future climate change is anticipated to modify these environmental parameters, potentially impacting the composition and dynamics of microbial communities.

## Author Contributions

**Gaëlle Bednarek:** data curation, formal analysis, investigation, validation, visualization, writing – original draft. **Hélène Agogué:** conceptualization, data curation, methodology, resources, supervision, validation, visualization, writing – original draft, writing – review and editing. **Arno Bringer:** conceptualization, funding acquisition, resources, writing – review and editing. **Tony Agion:** conceptualization, funding acquisition, resources, writing – review and editing. **Vincent Delafont:** conceptualization, data curation, methodology, supervision, validation, visualization, writing – original draft, writing – review and editing. **Yann Héchard:** conceptualization, data curation, funding acquisition, methodology, project administration, supervision, validation, writing – original draft, writing – review and editing.

## Conflicts of Interest

The authors declare no conflicts of interest.

## Supporting information


**Figure S1:** Monthly monitoring of environmental factors at the five sampling stations. The following correspond to: (A) salinity, (B) temperature, (C) dissolved oxygen and (D) precipitation.

**Figure S2:** Primer test against the PR2 primer database. Newly designed primers for Paramoeba (A) Naegleria (B) Vermamoeba (C) and Vannella (D) were tested against the PR2 primer database—v.2.0.0., allowing two mismatches maximum.

## Data Availability

Data sharing not applicable to this article as no datasets were generated or analysed during the current study.
