# Supplementary material for: Temporal and Spatial Survey on the Abundance of Amoebae and Bacteria in an Estuary and the Role of Environmental Parameters
Source: Environ Microbiol Rep. 2025 Sep 22;17(5):e70198. doi: 10.1111/1758-2229.70198 (PMC12454178; doi:10.1111/1758-2229.70198)
Supplement: Supplementary file 1 — Figure S1: Monthly monitoring of environmental factors at the five sampling stations. The following correspond to: (A) salinity, (B) temperature, (C) dissolved oxygen and (D) precipitation. [file EMI4-17-e70198-s001.pdf]

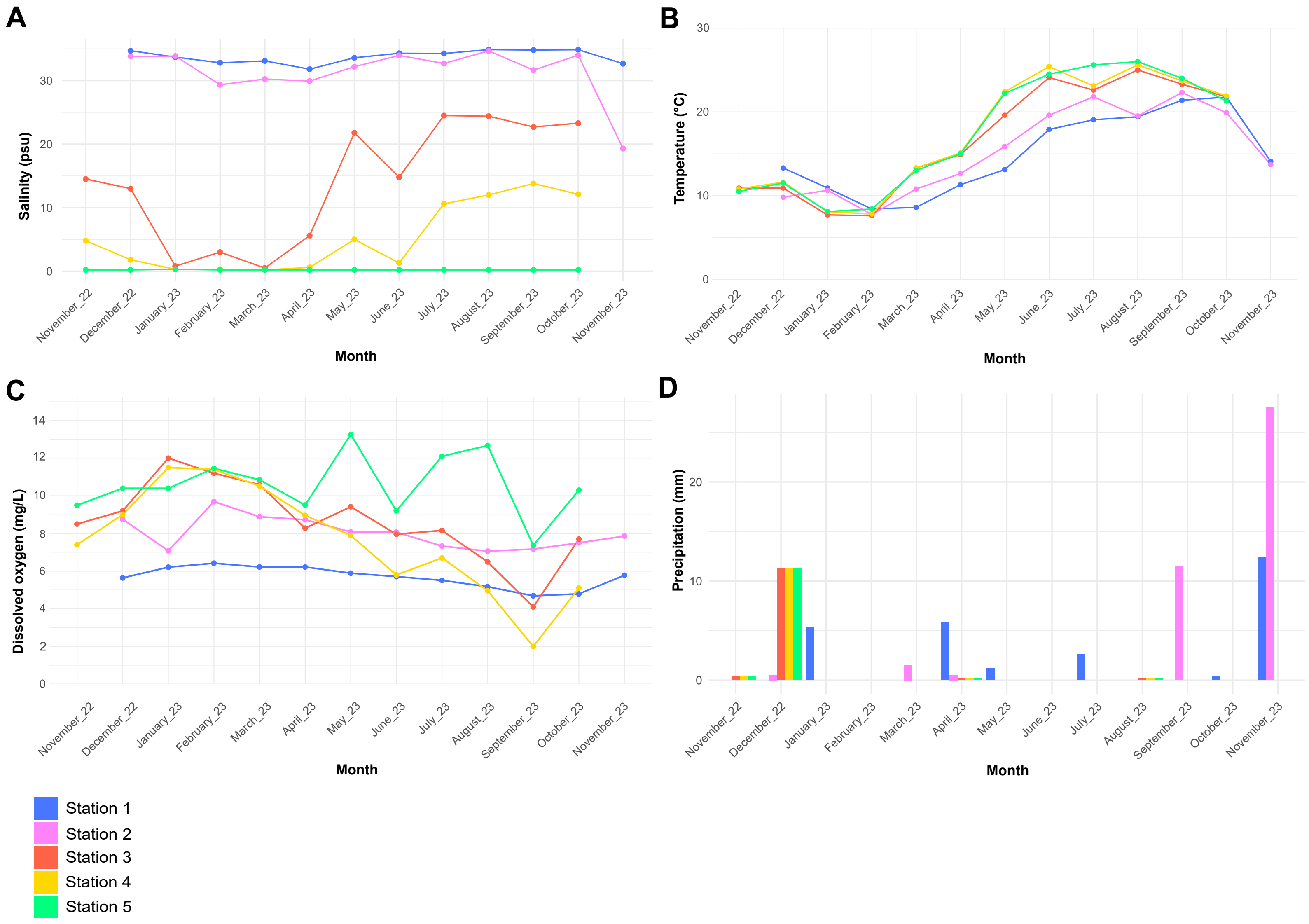

Supplementary information Figure 1. Monthly monitoring of environmental factors at the five sampling stations. The following correspond to: A) Salinity, B) Temperature, C) Dissolved Oxygen and D) Precipitation.
