# Supplementary material for: Temporal and Spatial Survey on the Abundance of Amoebae and Bacteria in an Estuary and the Role of Environmental Parameters
Source: Environ Microbiol Rep. 2025 Sep 22;17(5):e70198. doi: 10.1111/1758-2229.70198 (PMC12454178; doi:10.1111/1758-2229.70198)
Supplement: Supplementary file 2 — Figure S2: Primer test against the PR2 primer database. Newly designed primers for Paramoeba (A) Naegleria (B) Vermamoeba (C) and Vannella (D) were tested against the PR2 primer database—v.2.0.0., allowing two mismatches maximum. [file EMI4-17-e70198-s002.pdf]

A)

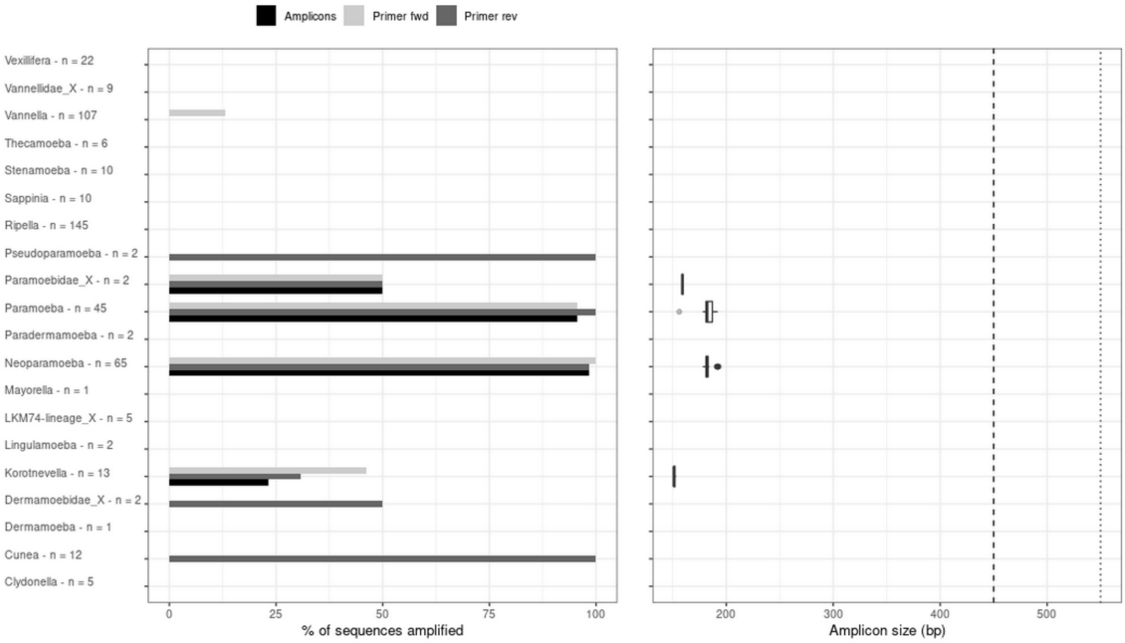

Amplicons
Primer fwd
Primer rev
Vexillifera - n = 22
Vannellidae_X - n = 9
Vannella - n = 107
Thecamoeba - n = 6
Stenamoeba - n = 10
Sappinia - n = 10
Ripella - n = 145
Pseudoparamoeba - n = 2
Paramoebidae_X - n = 2
Paramoeba - n = 45
Paradermamoeba - n = 2
Neoparamoeba - n = 65
Mayorella - n = 1
LKM74-lineage_X - n = 5
Lingulamoeba - n = 2
Korotnevella - n = 13
Dermamoebidae_X - n = 2
Dermamoeba - n = 1
Cunea - n = 12
Clydonella - n = 5
0
25
50
75
100
% of sequences amplified
200
300
400
500
Amplicon size (bp)

B)

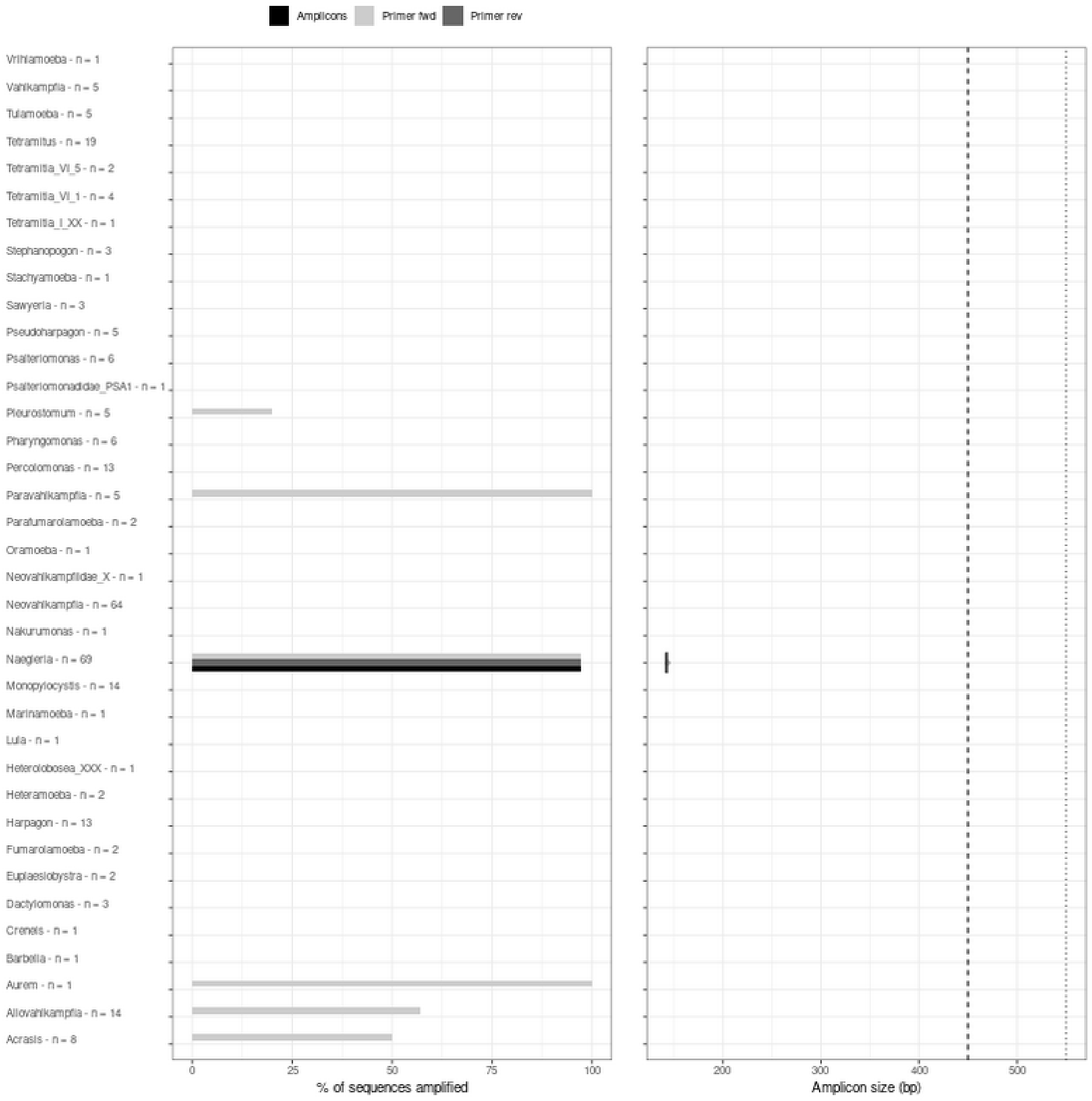

Amplicons
Primer fwd
Primer rev
Vrihiamoeba - n = 1
Vahlkampfia - n = 5
Tulamoeba - n = 5
Tetramitus - n = 19
Tetramitia_VI_5 - n = 2
Tetramitia_VI_1 - n = 4
Tetramitia_I_XX - n = 1
Stephanopogon - n = 3
Stachyamoeba - n = 1
Sawyeria - n = 3
Pseudoharpagon - n = 5
Psalteriomonas - n = 6
Psalteriomonadidae_PSA1 - n = 1
Pleurostomum - n = 5
Pharyngomonas - n = 6
Percolomonas - n = 13
Paravahlkampfia - n = 5
Parafumarolamoeba - n = 2
Oramoeba - n = 1
Neovahlkampfiidae_X - n = 1
Neovahlkampfia - n = 64
Nakurumonas - n = 1
Naegleria - n = 69
Monopylocystis - n = 14
Marinamoeba - n = 1
Lula - n = 1
Heterolobosea_XXX - n = 1
Heteramoeba - n = 2
Harpagon - n = 13
Fumarolamoeba - n = 2
Euplaesiobystra - n = 2
Dactylomonas - n = 3
Creneis - n = 1
Barbelia - n = 1
Aurem - n = 1
Allovahlkampfia - n = 14
Acrasis - n = 8
0
25
50
75
100
% of sequences amplified
200
300
400
500
Amplicon size (bp)

C)

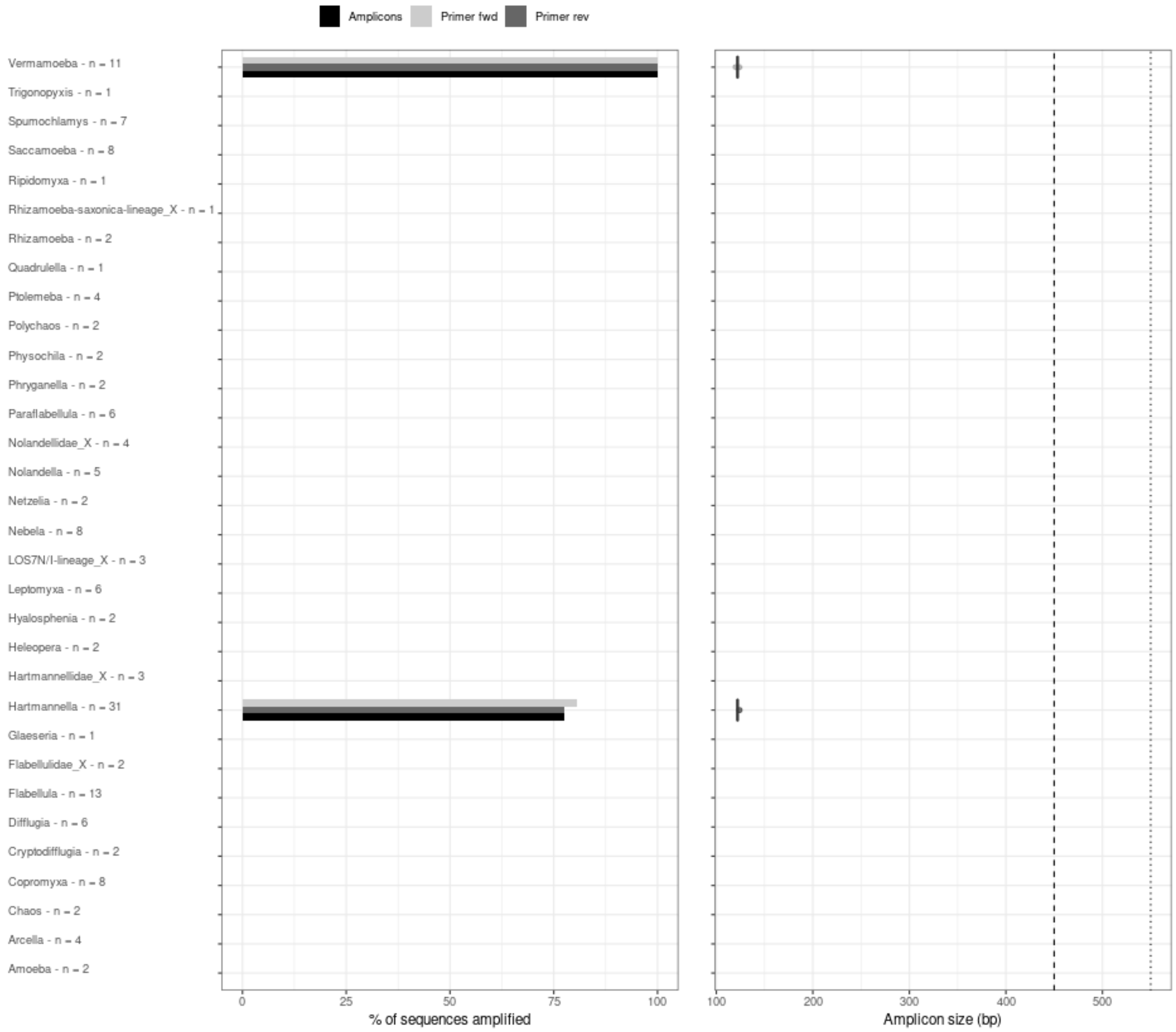
Amplicons
Primer fwd
Primer rev
Vermamoeba - n = 11
Trigonopyxis - n = 1
Spumochlamys - n = 7
Saccamoeba - n = 8
Ripidomyxa - n = 1
Rhizamoeba-saxonica-lineage_X - n = 1
Rhizamoeba - n = 2
Quadrulella - n = 1
Ptolemeba - n = 4
Polychaos - n = 2
Physochila - n = 2
Phryganella - n = 2
Paraflabellula - n = 6
Nolandellidae_X - n = 4
Nolandella - n = 5
Netzelia - n = 2
Nebela - n = 8
LOS7N/I-lineage_X - n = 3
Leptomyxa - n = 6
Hyalosphenia - n = 2
Heleopera - n = 2
Hartmannellidae_X - n = 3
Hartmannella - n = 31
Glaeseria - n = 1
Flabellulidae_X - n = 2
Flabellula - n = 13
Difflugia - n = 6
Cryptodifflugia - n = 2
Copromyxa - n = 8
Chaos - n = 2
Arcella - n = 4
Amoeba - n = 2
0
25
50
75
100
% of sequences amplified
100
200
300
400
500
Amplicon size (bp)

D)

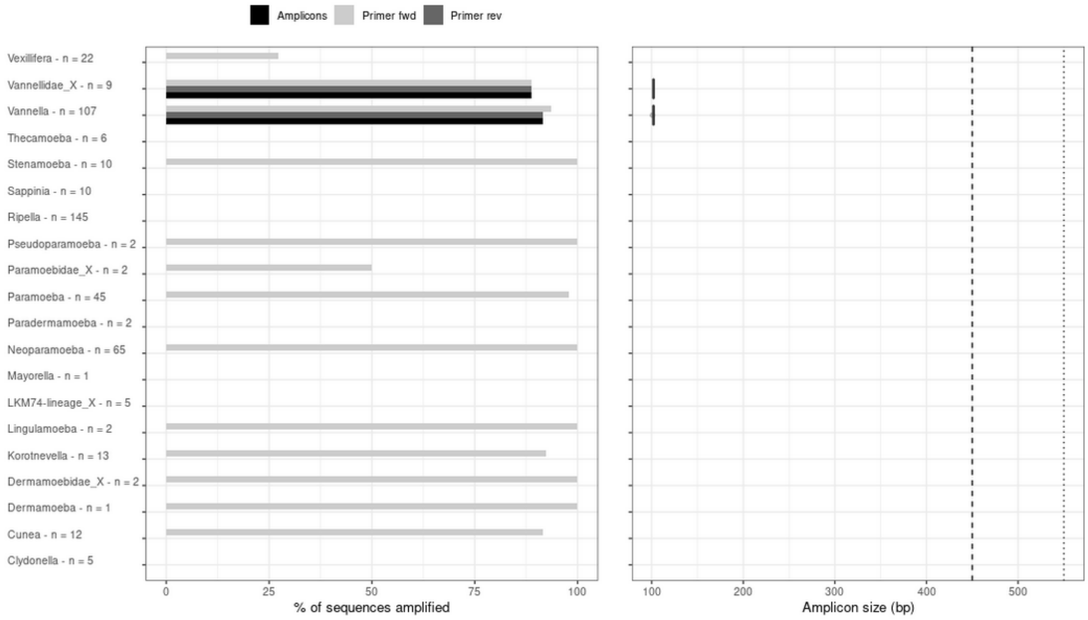

Supplementary information Figure 2. Primer test against the PR2 primer database. Newly designed primers for Paramoeba A) Naegleria B) Vermamoeba C) and Vannella D) were tested against the PR2 primer database – v.2.0.0., allowing two mismatches maximum.
